# Microstructure and Wear Behavior of WMoTaNbV Refractory High-Entropy Alloy Coating on Ti6Al4V Alloy Surface Prepared by Laser Cladding

**DOI:** 10.3390/ma18081770

**Published:** 2025-04-12

**Authors:** Jiazhu Liang, Hongxi Liu, Qinghua Zhang, Ling Zhou, Yuanrun Peng

**Affiliations:** Faculty of Materials Science and Engineering, Kunming University of Science and Technology, Kunming 650093, China; 19525852205@163.com (J.L.); zqhzam1001@163.com (Q.Z.); 17869418798@163.com (L.Z.); pyr6789@163.com (Y.P.)

**Keywords:** radiofrequency plasma spheronization, refractory high-entropy alloy, coating, laser cladding, wear behavior

## Abstract

WMoTaNbV refractory high-entropy alloys (RHEAs) have received widespread attention due to their excellent low-temperature toughness, hardness, and wear resistance. In recent years, the rapid development of surface modification technology represented by laser cladding has provided a new technological path for RHEA surface forming, and at the same time put forward higher requirements for raw material powder. In this study, WMoTaNbV RHEA spherical powder was prepared by radiofrequency plasma spheronization, and then WMoTaNbV RHEA coating was prepared on the surface of Ti6Al4V (TC4) alloy by laser cladding technique. The experimental results show that the prepared alloy powders have very high sphericity and are almost free of agglomeration and oxidation. Coatings with laser powers of 3.1–3.9 kW (gradient setting of 2 kW) were tested, with the 3.3, 3.5, and 3.7 kW coatings showing the best of the abrasion resistance. The microhardness of the 3.3 kW, 3.5 kW, and 3.7 kW coatings was 1.72, 1.97, and 1.76 times higher than that of the substrate, and the wear resistance was 1.83, 3.42, and 2.13 times higher than that of the TC4 substrate, respectively. This experimental result shows that the surface hardness and wear resistance of WNbMoTaV RHEA coating can be effectively improved by precisely regulating the laser power, thus improving the surface hardness and friction and wear properties of TC4 titanium alloy.

## 1. Introduction

Titanium alloys have many excellent properties such as high specific strength, low density, low flexural strength ratio, and good weldability, and are widely used as important structural materials in the fields of shipbuilding, the petrochemical industry, and the aerospace industry [1,2,3]. At present, the most widely used titanium alloy is Ti6Al4V (TC4) alloy. TC4 titanium alloy is a typical α + β dual-phase titanium alloy, which combines the excellent properties of α titanium alloy and β titanium alloy and, thus, has high practical value in aerospace, medical, and communication fields; however, the inherent disadvantages of TC4, such as low hardness and poor friction and wear properties, limit its application as a structural material in extreme service environments [4,5].

In most cases, damage when TC4 titanium alloy is used as a metal component material, tends to occur first on the surface. This damage is mainly due to the insufficient properties of titanium alloys such as low hardness and low wear resistance [6]. In order to improve the hardness and wear resistance of TC4 titanium alloy, it can be improved from two aspects. First, a variety of new titanium alloys with excellent properties can be developed from the material itself. However, doing so may change the composition and content of elements in titanium alloys, thus affecting their original advantages. Secondly, it is possible to start with surface treatment, using various surface modification techniques to obtain coating with high hardness and wear resistance on the surface of titanium alloys, thus improving the surface defects of titanium alloys while retaining their associated excellent properties. With the development of surface coating technology, the types of coatings are becoming more and more diverse. Composite coatings provide unique technological advantages for improving mechanical and tribological surface properties [7,8]. However, composite coatings nowadays still have problems such as uneven dispersion of coating composition and weak interfacial bonding. Therefore, it is crucial to develop and design coatings with relatively homogeneous compositions and strong bonding ability to the TC4 titanium alloy substrate.

Typically, the interface between the TC4 substrate and coating with weak bonding strength is prone to interfacial defects due to insufficient wettability, forming stress concentrations and, thus, reducing the mechanical properties and stability of the coating [9]. The coating prepared by traditional surface modification techniques such as thermal spraying and thermal plating are weak and have poor bonding with the substrate, making it difficult to meet the usage requirements of components working under high-contact stress [10,11]. As a new surface modification technology, laser cladding has the advantages of high-energy density, fast melting and solidification speed, and controllable coating thickness [12]. The materials used for laser cladding to modify the surface of titanium alloys are mainly metals, alloy powders, and some ceramic materials that match the thermophysical properties of the substrate [13]. Since the laser cladding process on the surface of titanium alloys is highly susceptible to the production of intermetallic compounds, it can lead to a sharp increase in residual stress in the coating, resulting in defects such as cracks and crazing in the coating. This is also one of the biggest pain points in laser cladding of titanium alloys at present [14]. Therefore, exploring more suitable cladding materials based on optimizing the best process is the key to solving this pain point.

High-entropy alloys (HEAs) are a new class of materials that have been rapidly developed in recent years, and have received widespread attention since they were proposed by Yeh [15] in 2004 due to their excellent low-temperature toughness, thermal stability, wear resistance, corrosion resistance, and other properties [16,17]. According to the different constituent elements, high-entropy alloys can be categorized into refractory high-entropy alloys, 3D transition group high-entropy alloys, lanthanide transition metal elements, and lightweight high-entropy alloys, etc. [18]. Among them, refractory high-entropy alloys (RHEAs) are a high-entropy alloy material composed of high-melting-point metal elements such as W, Mo, Ta, Nb, V, Ti, Cr, Hf, Zr, and so on as the main element, or an additional small amount of B, Si, Al, and so on [19]. Zhang [20] found that the slow diffusion effect and high-entropy effect of RHEA were more obvious, which reduced the free energy of the system to a larger extent and formed a relatively stable microstructure. It has been shown that laser cladding of RHEA on the surface of titanium alloy can improve its surface hardness, wear resistance, and other properties [21,22]. Currently, MoNbTaW-based RHEAs are the more widely used RHEAs. Ren [23] prepared NbMoTaWTi RHEA coating on the surface of TC4 alloy using laser melting, and the coating showed a single BCC phase. The microhardness of the coating was increased by 72% over the substrate and the wear resistance was increased by 121.9% over the substrate. Hao [24] prepared MoNbTaWTi (Si_3_N_4_)_x_ (x = 0.5, 1.0, 1.5 and 2.0) RHEA composite coatings on the surface of TC4 alloys using laser cladding. The experimental results show that the coatings were mainly composed of BCC phase and TiN/(Nb, Ti)_5_Si_3_ ceramic particle phase. The high-strength BCC phase resulted in a much higher microhardness and wear resistance of the coatings. Senkovet [25] achieved the first preparation of two RHEAs, WMoTaNb and WMoTaNbV, using a vacuum arc melting technique. It was found that both alloys possessed a simple high-strength BCC phase and significant solid solution strengthening. In addition, it was shown that the reduction in grains and cracks in the WMoTaNbV RHEA indicates that the addition of the V element refines the alloy grains and reduces the tendency of crack generation, thus improving the properties of the alloy. Given that the WMoTaNbV RHEA possesses excellent properties, WMoTaNbV RHEA will be used as the laser cladding material in this experiment.

Preparation of high-quality RHEA powder is essential for the preparation of RHEA coating by laser cladding. Currently, RHEA powder is prepared by mechanical alloying, plasma rotating electrode atomization, and radiofrequency plasma spheronization. Compared with mechanical alloying, plasma rotating electrode atomization, and other methods, radiofrequency plasma spheronization has the characteristics of high heat, high enthalpy, adjustable atmosphere, and no electrode contamination, which is especially suitable for the preparation of high-melting-point metal spherical powder. Liu [26] successfully prepared spherical WMoTaNb RHEA powder with low impurity content, better fluidity, and narrower particle size distribution by using a technological path combining spray drying and radiofrequency plasma spheronization. After radiofrequency plasma spheronization, the microhardness of the powder reached 10.09 GPa. Tae-Wook Na [27] prepared TaNbHfZrTi RHEA spherical powder by melting, hydrogenated dehydrogenation crushing, and plasma spheronization. After hydrogenation, the alloy was completely transformed from the BCC phase to the M_3_H_2_ metal hydride phase, which increases the brittleness and can be crushed into irregular fine powder. After plasma spheroidization, the alloy powder reverts to a single solid solution BCC phase. Xia [28] prepared WTaMoNbZr RHEA alloy powder by a process of melting, hydrogenation, crushing, and radiofrequency plasma spheroidization. The powder spheroidization rate was 95.3% with an average particle size of 37.5 µm, and the phase structure after spheroidization consisted of a single high-strength BCC phase, which led to a nano-hardness of 7.99 GPa.

Synthesizing the previous studies, it is known that WNbMoTaV RHEA has good microhardness and wear resistance. In view of this, in this paper, ball milling was used to crush the mixed monolithic elemental powders, then combined with spray granulation to prepare spherical precursor powders, and radiofrequency plasma spheronization was used to prepare spherical RHEA powder, in order to obtain WNbMoTaV RHEA powder with better sphericity and smaller particle size distribution. Finally, the powder was applied to the laser cladding process on the surface of titanium alloy, aiming to improve the surface hardness and wear resistance of TC4 titanium alloy, so as to prolong the service life of titanium alloy in extreme environments and to broaden the scope of its use.

## 2. Materials and Methods

### 2.1. Experimental Materials and Preparation of Coating

Five kinds of powders with purity of 99.5% W, Mo, Ta, Nb, and V (Guangdong Chengfeng Material Technology Co., Ltd., Foshan, China) were calculated and weighed according to the equimolar atomic ratio. Due to the large difference in particle size between the five powders, the powders were first crushed and mixed by mechanical alloying. The ball milling jars lined with PTFE and agate grinding balls were used as ball milling media to crush and mix the powders, and the ball milling jars were evacuated and filled with argon before ball milling operation, which was repeated for three times to ensure that the ball milling process was in an inert gas-protected environment. The experimental parameters were as follows: ball milling speed of 350 r·min^−1^, ball to material ratio of 5:1, and 10 h of ball milling. Starch (2.5 wt.%) was chosen as the binder for the granulation experiment, and the solid content was set to 40 wt.%, then the starch was added into deionized water and put into a thermostatic thermostat, and heated to above 70 °C while stirring to make the starch dissolve in the deionized water. Then the mixed powder, starch aqueous solution, and stainless steel ball were put into the stainless steel ball mill tank for wet grinding, and the wet grinding parameters were set as follows: ball mill rotational speed of 350 r·min^−1^, ball material ratio of 4:1, ball milling time of 3 h. After the slurry preparation through the pelletizing equipment (Shanghai Hefan Instrumentation HF-5L Centrifugal Spray Dryer, Shanghai, China), which used a peristaltic pump to pump the slurry into the pelletizing equipment nebulizer spray pelletizing, the entire spraying experimental equipment parameters were set as follows: inlet air temperature 180 °C, outlet air temperature 110 °C, peristaltic pump 450 mL·h^−1^, nebulizer frequency 250 Hz. Finally, the previously prepared WNbMoTaV RHEA pelletized powder was sphericalized to obtain spherical WNbMoTaV powder using a radiofrequency plasma spheronization powder-making system (TEKNA, Sherbrooke, QC, Canada, 40 kW) with the process parameters set as follows: spheronization power of 35 kW, and sheath gas composition of Ar:H_2_ (70 L·min^−1^:6 L·min^−1^). After the spheronization process was completed, in order to remove the ultrafine particles that may be attached to the surface of the powder, the experiment was carried out using JP-080S ultrasonic cleaning machine (Shenzhen Jiemeng Technology Co., Shenzhen, China), which uses ethanol as the cleaning agent for ultrasonic cleaning. The sonic action of the ultrasonic cleaner can effectively remove the dirt and attached particles on the powder surface, thus ensuring the purity of the powder and the quality of subsequent applications.

**Figure 1:**
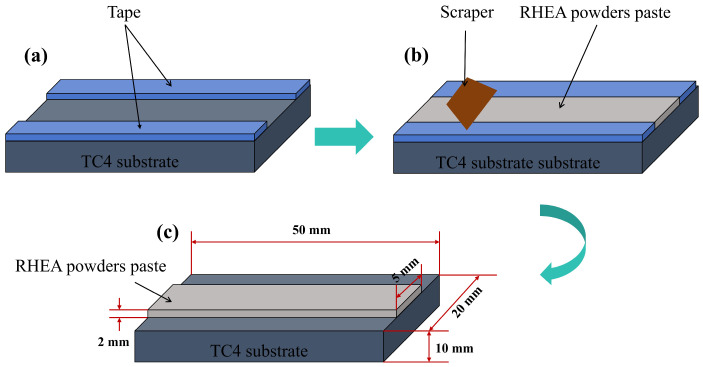
Schematic of powder paste coating process: (**a**) fixing the tape; (**b**) spreading the powder and smoothing it with a spatula; (**c**) removing the tape.

In this study, TC4 titanium alloy (specimen size 50 mm × 20 mm × 10 mm) produced by Beijing R&B Co. (Beijing, China). was used as the substrate material. One side of each TC4 substrate sample was polished with 400-grit and 600-grit SiC sandpaper to remove surface oxides and stains, which also helped to reduce laser reflection. Then the WMoTaNbV RHEA powder was preset on the TC4 substrate using the presetting powder method as shown in Figure 1. Firstly, a 2 mm thick tape was pasted on the TC4 alloy plate, and then a gap of 5 mm was left in the notch, and the mixed powder was spread on this gap and smoothed with a scraper. Finally, the plate was placed in a drying oven at 50 °C for 12 h, after which the tape was removed. Through this pre-setting process, the fusion powder can be evenly distributed on the substrate, which provides a good foundation for the subsequent laser fusion process.

In this experiment, a fiber laser (MFSC-6000) (Shenzhen Chuangxin Laser Co., Shenzhen, China) equipped with 6000 W was used to melt WNbMoTaV RHEA coating on TC4 substrate. The optimized laser cladding process parameters were as follows: laser spot diameter was set at 5 mm, scanning speed at 600 mm·min^−1^, and defocusing amount at 20 mm. To prevent oxidation during the laser cladding process, helium was used as a protective gas (99.99% purity, blown to one side of the melt pool) with a gas flow rate of 15 L·min^−1^. For the preparation of laser cladding WNbMoTaV RHEA coating, a laser power of 3.1–3.9 kW (with a gradient setting of 2 kW) was selected in order to investigate the effect of the laser power on the laser cladding coating.

### 2.2. Microstructure Characterization and Performance Test of RHEA Coating

The phase composition of the laser-fused WNbMoTaV RHEA coating was characterized using a D/max-3BX X-ray diffractometer (Rigaku Corporation, Tokyo, Japan) (CuKα, λ = 1.54056 nm) at an operating voltage of 40 kV and an operating current of 30 mA. The scanning range was 2θ 10°~90° with a scanning step of 5° min^−1^ (CSM, continuous scanning mode). A scanning electron microscope (SEM) of the Czech company TESCAN, model VEGA 3 GMU (TESCAN, Brno, Czech Republic), and its accompanying Oxford detector-type energy spectrometer (Oxford Instruments, Oxford, England) (energy-dispersive spectroscopy (EDS)) were used to analyze the cross-sectional morphology of the powder and coating specimens, microstructure, and elemental composition of the powder and coating specimens, as well as subsequent morphological observation of abrasion marks on the coating and substrate surfaces, and quantitative analysis of the composition of individual samples.

The hardness of the cross-section of the coating sample was measured using a fully automated Vickers microhardness tester of the model HMV-G-FA (Shimadzu Corporation, Kyoto, Japan). During the experiment, the applied test force was set at 0.98 N, and the force was held to act continuously for 10 s. The friction and wear properties of the materials were tested on a Bruker (CETR) type UMT-2 (Bruker Nano Surfaces, Billerica, MA, USA) friction and wear tester. The friction mode was linear reciprocating motion, the friction partner was Si_3_N_4_ ceramic ball (4 mm in diameter), the length of abrasion mark was 2 mm, the load was 20 N, the oscillation rate was 1 Hz, and the linear reciprocating time was 30 min, and the 3D shape of the wear marks was determined using a Rtec-3D three-dimensional profiler (Rtec Corporation of America, San Jose, CA, USA). At the end of the friction wear test, the wear morphology of the sample was analyzed by SEM, and the results of the SEM analysis will provide a comparison of the wear surface morphology of the coating and the substrate during friction wear in order to determine the wear mechanism in the specimen.

## 3. Results

### 3.1. Microstructure of the Powders

**Figure 2:**
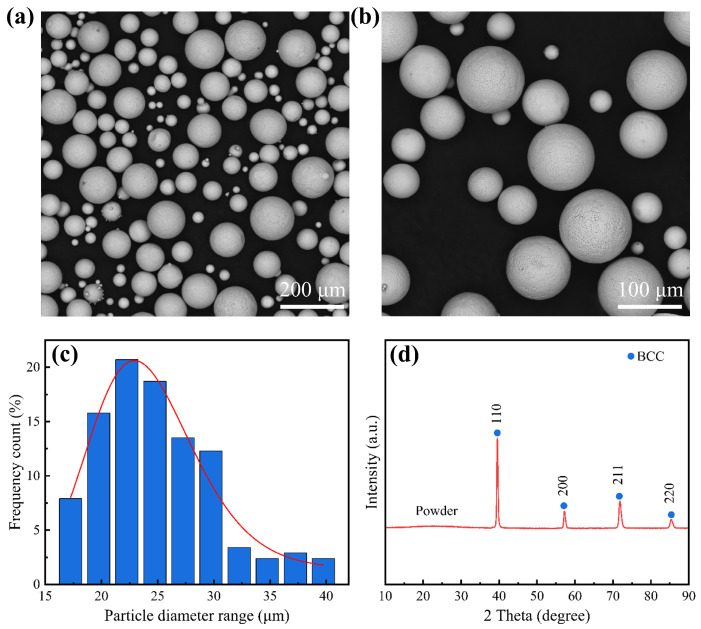
Microscopic morphology, particle size distribution, and XRD spectra of WNbMoTaV RHEA alloy powder: (**a**) SEM image of the alloy powder; (**b**) magnified SEM image of the alloy in Figure (**a**); (**c**) particle size distribution of the alloy powder; (**d**) XRD pattern of the alloy powder.

Precise control of starch content during slurry formulation is important to optimize the quality and properties of pelletized powder. Appropriate starch content helps to form a uniform and well-sphericalized powder during the granulation process. Similarly, different radiofrequency plasma power and sheath gas atmospheres are crucial for the morphology of WNbMoTaV powder obtained by spheronization, and suitable power and sheath gas atmospheres can obtain alloy powder with higher sphericity. Figure 2a–c show the morphology of granulated powder with starch content of 2.5 wt.% and solid content of 40 wt.%. Observation of the SEM images of Figure 2a–c show that the alloy powder is relatively homogeneous and the prepared alloy powder has a high degree of sphericity. Therefore, when starch was used as binder and its composition was precisely controlled at 2.5 wt.% (40 wt.% solids), the pelletized powder showed a high degree of homogeneity. During the spheronization process, when the plasma power was set to 35 kW and the sheath gas ratio was precisely controlled to be 70 L·min^−1^:6 L·min^−1^ for Ar:H_2_, no unmelted or unspheronized particles were observed, and the spheronization rate of the powder was close to 100%. In addition, not only was it observed that the prepared alloy powder had a good sphericity, but it was also observed that the alloy powder had no obvious fragmentation, only a small amount of agglomeration, and no oxidation phenomenon occurred.

The particle size distribution of the granulated powder was analyzed; as shown in Figure 2c, the particle size distribution of the alloy powder was 15–45 μm, and the particle size distribution was narrow. The XRD pattern of WNbMoTaV refractory high-entropy alloy powder is shown in Figure 2d. From Figure 2d, it can be seen that the WNbMoTaV refractory high-entropy alloy powder consists of a single BCC solid solution phase, and no other phases are generated. This lays the foundation for the subsequent laser cladding to prepare coating with simple phase structure. The prepared WNbMoTaV RHEA powder was subjected to EDS surface scanning to determine the compositional distribution of the alloy, which is shown in Table 1.

### 3.2. Surface Morphology and Microstructure of Coating

**Figure 3:**
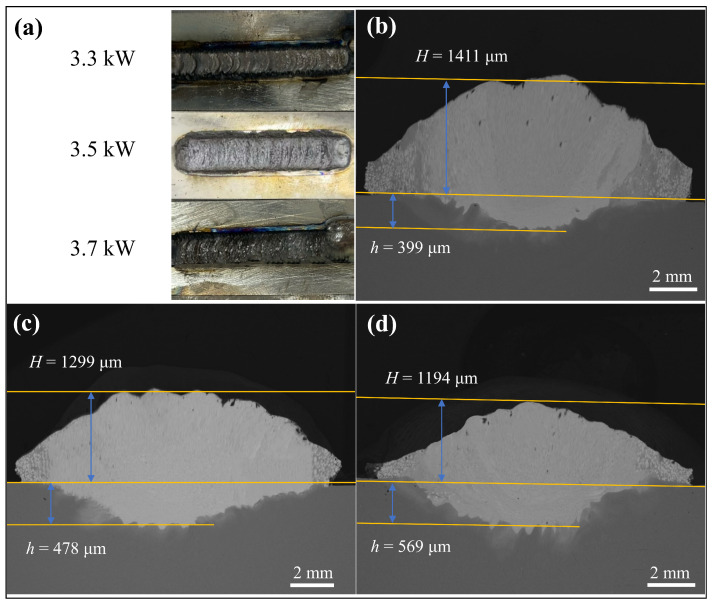
Macroscopic morphology and cross-sectional morphology of WNbMoTaV RHEA coatings at different laser powers: (**a**) macroscopic morphology of the coating; (**b**) 3.3 kW; (**c**) 3.5 kW; (**d**) 3.7 kW.

Of the coatings prepared at laser powers of 3.1–3.9 kW (with a gradient setting of 2 kW), the quality of the coatings at 3.1 kW and 3.9 kW was poorer, and, therefore, these two coatings will not be subsequently analyzed. Figure 3a shows the macroscopic morphology of the WNbMoTaV coating at a scanning speed of 10 mm∙s^−1^, a spot diameter of 5 mm, and different powers (3.3 kW, 3.5 kW, and 3.7 kW). The surfaces of all three coatings at different powers show a continuous and homogeneous condition without porosity and obvious macroscopic cracks, which indicates that the coatings were well formed. Non-melting alloy powder can be found for coating with a laser power of 3.3 kW, probably due to the low laser power, which resulted in a slightly lower laser energy density and a failure to completely melt the alloy. The macroscopic coating quality of the coating with a laser power of 3.5 kW was good, no obvious non-melting powder was seen, and the coating showed a clear metallic luster. The macroscopic morphology of the coating with a laser power of 3.7 kW does not show any obvious non-melting alloy powder, and the coating has a good macroscopic quality, but the coating is darker, which is due to the high laser power, resulting in a partial over-burning of the coating.

The cross-sectional morphology of the three coatings is shown in Figure 3b–d. The cross-sectional microstructures of the WNbMoTaV RHEA coatings can be divided into coating, bonding zone, and matrix parts. From the cross-sectional morphology of the coatings, it can be seen that the maximum thickness of the coatings is about 1.7 mm and the width is about 6 mm, the coatings are dense without obvious cracks and holes, and the coatings have good metallurgical bonding with the substrate, which indicates that the quality of the prepared coatings is good. It is worth noting that when the laser power was 3.3 kW, the coating produced a small amount of porosity in the fused coating due to the lower laser power used. Since the WNbMoTaV RHEA is mainly composed of high-melting-point elements, the coating still has a small amount of non-melting WNbMoTaV alloy powder particles. The volume percentage of insoluble powder in the coatings was analyzed by Photoshop software (Version number: v26.5.0), and the ratio of non-melting alloy powder for coatings with laser powers of 3.3 kW, 3.5 kW, and 3.7 kW were 19.3%, 11.2%, and 7.8%, respectively. It can be observed that the coating insolubility of the alloy powder decreases significantly as the laser power increases. This is due to the fact that the increase in laser power leads to an increase in laser energy density, and the laser energy irradiated on the surface of the alloy powder can melt the alloy powder more comprehensively. Usually the dilution rate can be used as an important index to evaluate the formability of high-entropy alloy coating [29], and the dilution rate can be calculated by using the area method. Its expression is shown in the following equation:

(1) $$\lambda =\frac{h}{H+h}\times 100\%$$

where *H* is the distance from the highest point of the coating to the surface of the substrate (μm); *h* is the distance from the surface of the substrate to the maximum melting depth of the coating (μm).

The properties of a coating are directly affected by the level of dilution rate. At higher dilution rates, the substrate material can significantly dilute the coating, which not only weakens the properties of the coating itself, but also increases the likelihood of cracking and deformation. On the contrary, if the dilution rate is too low, the interface between the coating and the substrate finds it difficult to realize a good bonding of gold, which makes it easy for the coating to fall off. Therefore, the key to obtain high quality coatings is to control the dilution rate. Based on the information shown in Figure 3a–c, the coating dilution rates at different laser powers were calculated to be 22.04%, 26.90%, and 32.27%, respectively. Obviously, the higher the laser power, the higher the dilution rate.

**Figure 4:**
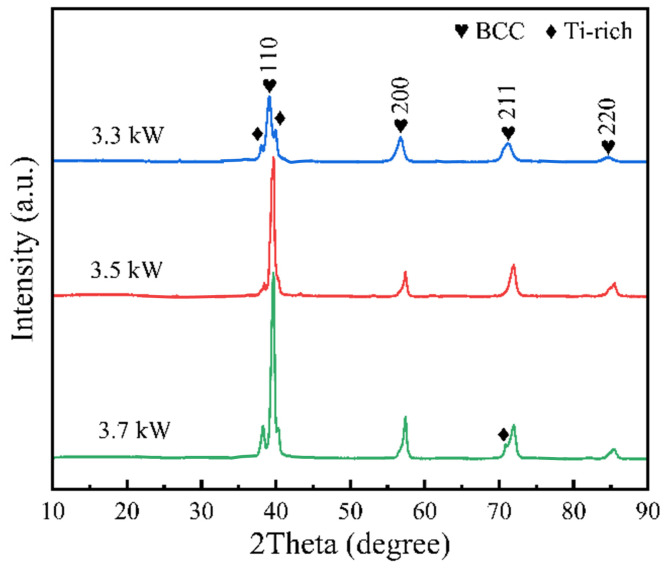
XRD pattern of WNbMoTaV RHEA coating.

Figure 4 shows the XRD pattern of the WNbMoTaV RHEA coatings at three laser rates. In order to determine the coating physical phase structure, it is necessary to derive and calculate based on Bragg’s equation shown in Equations (2) to (3) below:

(2) 2dsinθ=nλ

where *d* is the crystal plane spacing (nm), *θ* is the angle between the incident light and the crystal plane (°), and *n* is the wavelength of the diffracted light (nm).

The cubic structure crystal spacing equation is:

(3) $$d=\frac{a}{\sqrt{h^{2}+k^{2}+l^{2}}}$$

where *a* is the lattice constant (nm) of the crystal, and *h*, *k*, and *l* are the crystallographic indices of the crystal.

Based on Equations (2) and (3), the formula for cubic crystals can be derived as shown in Equation (4):

(4) $$sin2\theta =\frac{\lambda^{2}}{4a^{2}}(h^{2}+k^{2}+l^{2})$$

The lattice type of the crystal can be determined by calculating the *sin*2*θ* value and performing successive ratios. According to the lattice extinction law, it can be seen that the crystal structures of the alloy are BCC and FCC structures for *sin*2*θ* ratios of 1:2:3:4: … or 3:4:8:11: …, respectively. From the XRD patterns shown in Figure 4, it can be seen from the XRD patterns of the three coatings according to the 2*θ* values of the diffraction peaks are 39.62°, 57.36°, 72.03°, and 85.19°, respectively, and the ratio of their sin2*θ* is about 1:2:3:4. Combined with the relative intensities of the diffraction lines at the different diffraction angles and the crystalline structures of the elements, it is possible to determine that these peaks are BCC phase diffraction peaks. In addition, comparing the standard PDF cards, the diffraction peaks at 38.19° and 40.27° are considered to be Ti-rich phases. In addition, the peak of the Ti-rich phase was found at 70.88° with a laser power of 3.7 kW. Therefore, the structure of the prepared coating phase consists of a single BCC phase and a Ti-rich phase.

It is noteworthy that the diffraction peaks become sharpened as the laser power increases to 3.5 kW. This is due to the increased crystallinity of the coating at a laser power of 3.5 kW and the fact that the coating is mainly composed of a single BCC phase with a simpler physical phase composition. The diffraction peaks of the Ti-rich phase generated in the coating were enhanced as the laser power was increased to 3.7 kW, indicating that more Ti-rich phase was generated in the coating. In addition, it should be noted that the intensities of the (110) and (211) peaks of the WNbMoTaV RHEA coating increase with increasing laser power, indicating that the coating undergoes a selective orientation with increasing laser power. As the laser power increases, the accumulated internal stresses and strain energy no longer dominate the preferential orientation, but the surface energy does, and the grains of the WNbMoTaV RHEA coating grow along the (110) direction with the lowest surface energy. So as the laser power increases, the grains in the coating grow along the (110) direction which has the lowest surface energy and fine grain structure, and the crystallinity of the coating increases. This also shows that there is a tendency to improve the crystallinity of the coating by adjusting the laser power.

Based on the measured XRD pattern, we can determine the grain size. The grain size was measured using the Scherrer equation [30]:

(5) $$\tau =\frac{0.9\lambda }{B\cos\theta }$$

where *τ* is the average grain size, *λ* is the X-ray wavelength, *B* is the line spread at half the maximum intensity in radians (FWHM), and *θ* is the diffraction angle.

Overall, the grain size of the coating decreases from 21.55 nm to 16.48 nm when the laser power is increased from 3.3 kW to 3.5 kW, and the grain size of the coating is 16.17 nm when the laser power is further increased to 3.7 kW. Higher laser power leads to smaller grain size.

**Figure 5:**
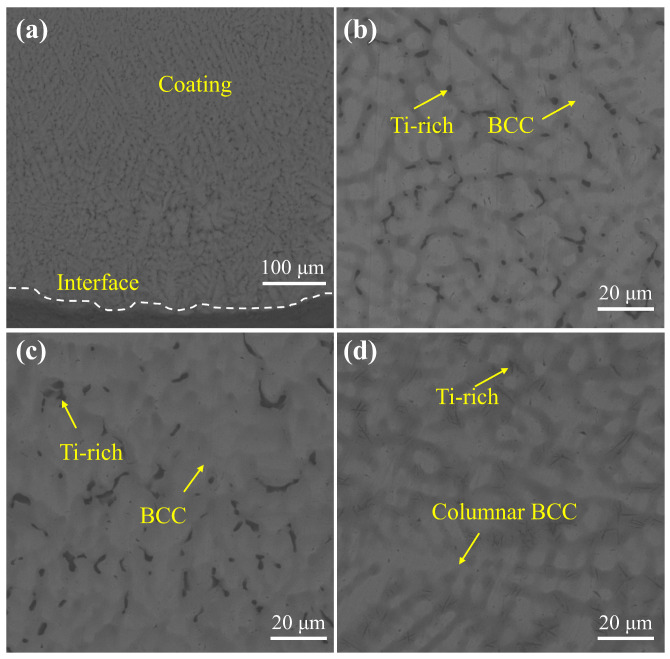
SEM images of the cross section of the WNbMoTaV RHEA coating at a laser power of 3.7 kW: (**a**) overall SEM pattern of the coating; (**b**) SEM pattern of the top of the coating; (**c**) SEM pattern of the middle of the coating; (**d**) SEM pattern of the bottom of the coating.

In terms of SEM microscopic morphology, the coating with a laser power of 3.7 kW was chosen as a typical example for illustration since the morphology of the coating was similar at the three powers, but the 3.7 kW coating had more phase groups. As shown in Figure 5a, the interface between the TC4 substrate and the WNbMoTaV coating has no obvious cracks, and the interface line is jagged. The metallurgical bond between the WNbMoTaV coating and the TC4 substrate is formed, which is conducive to a more adequate occlusion of the two materials, thus improving the interfacial bond strength. From the top of the coating to the substrate part, the microstructure morphology shows a clear delamination phenomenon, and this morphological feature can be interpreted as typical of the laser melting process, which is characterized by both fast melting and fast solidification. During the laser cladding process, due to the instantaneous high temperature and rapid cooling, the cooling rate can reach the level of 104–106 K·s^−1^. Therefore, when the alloy starts to solidify, the resulting temperature gradient (G_L_) is extremely large and non-homogeneous nuclei are formed at the solid–liquid interface. These nuclei expand outward centered on the solid–liquid interface and form a layer of granular planar crystals upon solidification. As the solid–liquid interface continues to advance, the solidification rate (R) increases and the G_L_/R ratio decreases, resulting in a gradual evolution of the interface from the original planar to an uneven columnar crystal organization.

Figure 5b–d show the enlarged images of the top, middle, and bottom of the coating. According to the XRD results, no new phases were formed in the coating except for the BCC phase and a small amount of Ti-rich phase generation, indicating that the white and gray areas can be considered as the BCC phase. At the top and middle of the coating, white BCC phases in the form of cells and petals, as well as Ti-rich phases interspersed at grain boundaries can be observed. As it extends down to the bonding zone, there is a longer white columnar crystal organization at the bottom, as well as interstitial dendritic Ti-rich phases at the grain boundaries, while there is a significant increase in the grey BCC phase region. Finally, it reaches the junction between the coating and the substrate, where a good metallurgical bond is formed between the coating and the substrate. Among them, the Ti-rich phase composition is higher at a laser power of 3.7 kW, which is consistent with the XRD results. This is due to the fact that during laser cladding, the laser beam induces molten pool stirring and the Marangoni effect [31], where the matrix phase diffuses into the coating and generates a Ti-rich phase with the coating elements, which is a typical phenomenon of laser cladding. However, if the laser cladding power is too high, the laser beam will cause strong stirring and stronger Marangoni effect, which will cause more matrix phase to diffuse into the coating, and more Ti-rich phase will be generated. Therefore, reasonable control of the laser power is crucial to be able to obtain a single BCC phase with high intensity.

**Figure 6:**
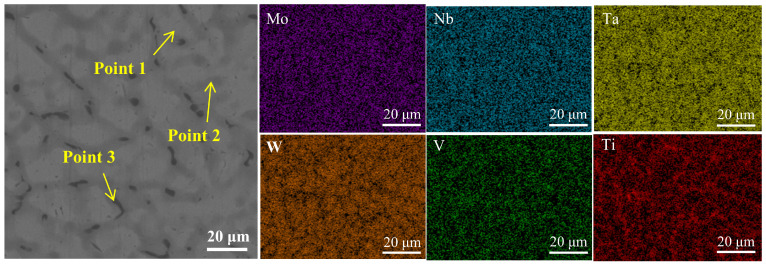
Energy dispersion spectrum (EDS) of WNbMoTaV RHEA coating at laser power of 3.7 kW.

In order to further analyze the elemental distribution of the BCC phase and the Ti-rich phase between the dendrites, EDS surface scanning was performed on the coating with a laser power of 3.7 kW; the surface scanning image is shown in Figure 6, and the results of the labeled points in the SEM image are listed in Table 2. According to the EDS face scanning results and the data in Table 2, it can be seen that the high-melting-point elements Ta and W are preferentially solidified and aggregated in the bright white region; the elements Mo, Nb, and V tend to be aggregated in the gray region; and the element Ti tends to be biased between the dendrites, and then diffuse into the interior of the crystal.

### 3.3. Microhardness and Wear of Coating

**Figure 7:**
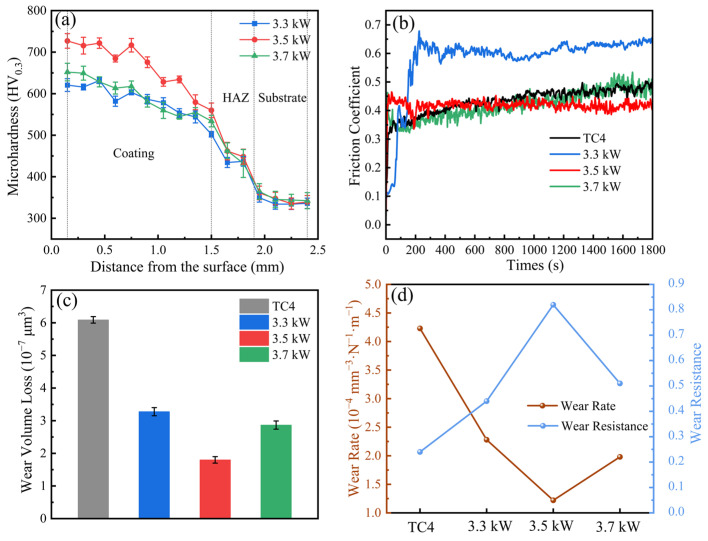
Frictional wear results of WNbMoTaV RHEA coatings on TC4 substrate and different laser powers: (**a**) microhardness; (**b**) friction coefficient; (**c**) wear volume; (**d**) wear rate and wear resistance.

Figure 7a shows the results of microhardness testing of WNbMoTaV RHEA coating cross-section. The curved hardness is divided into three zones, which are coating zone, heat-affected zone, and matrix zone. The average microhardness values of the 3.3 kW, 3.5 kW, and 3.7 kW coatings and the TC4 substrate were 586.71 HV_0.3_, 670.67 HV_0.3_, 601.22 HV_0.3_, and 341.25 HV_0.3_, respectively. The hardnesses of the 3.3 kW, 3.5 kW, and 3.7 kW coatings were 1.72, 1.97, and 1.76 times higher than the substrate, respectively. The results show that the WNbMoTaVRHEA coating significantly increased the microhardness of the TC4 substrate surface. This is mainly attributed to the increase in lattice distortion energy in the single-phase BCC solid solution structure, which led to the enhancement of solid solution, strengthening of the coating during the laser melting process [32]. The microhardness profile of the coatings conforms to the typical microhardness profile of laser- melted cladding layers, with coatings close to the substrate having a lower microhardness than coatings far from the substrate, but still higher than coatings on the substrate. This can be attributed to two main factors: first, the rapid melting and cooling during the laser cladding process prevents the grains in the cladding from growing rapidly, which leads to significant differences in the organization of the upper and lower portions of the cladding; in addition, when the coating is closer to the substrate, it has a higher dilution rate, and the dilution of the coating by the substrate will result in a gradient in the microhardness of the coating [33]. At the same time, the dilution effect of laser cladding leads to the formation of a transition zone where the cladding material and the substrate material coexist, making the hardness of the transition zone exactly between the high hardness of the coating zone and the low hardness of the substrate.

A laser power of 3.3 kW resulted in a low microhardness of the coating because the laser power was too low, the coating had the highest percentage of unfused alloy powder of the three coatings, and the low laser power resulted in an increase in surface defects, so it resulted in a low microhardness of the coating. When the laser power was increased to 3.5 kW, the coating microhardness was the highest. This is mainly because the surface quality of the coating is the best, and the quality of the generated high-strength BCC phase is the best; in addition, when the laser power is increased, the grain size of the coating decreases, and the microhardness of the coating is elevated by the fine-grained strengthening, so the coating has the highest microhardness. When the laser power is further increased to 3.7 kW, the microhardness of the coating decreases, which is mainly due to the following reasons: firstly, due to the laser power is too large, inducing the coating overcooking phenomenon, resulting in a decrease in the surface quality of the coating; secondly, when the laser power is 3.7 kW, the coating dilution rate is relatively high, and more matrix phase diffuses into the coating, and the microhardness of the matrix phase is low, resulting in a lower microhardness of the coating.

Figure 7b–d show the friction and wear test results of WNbMoTaV RHEA coating and TC4 substrate. The coefficient of friction (COF) curve is shown in Figure 7b, where the friction coefficient of the coating is higher than that of the substrate for a laser power of 3.3 kW; the friction coefficients of the coating and the substrate are about the same for laser powers of 3.5 kW and 3.7 kW. Under the same sliding conditions, the friction process can be divided into two stages: the early-wear stage and the stable-wear stage. Both the TC4 substrate and the coatings with laser powers of 3.3 kW, 3.5 kW, and 3.7 kW exhibit a sharp increase in the coefficient of friction for a very short time at the beginning of the friction, followed by stabilization. Significant changes in the friction coefficient of RHEA during the friction process can be attributed to the microhardness of the alloy, the phase composition, the organizational homogeneity, and the generation of cracks on the wear surface. In the steady-wear stage, the coefficient of friction of the coating with a laser power of 3.5 kW is relatively stable without significant fluctuations; the coefficient of friction curve of the coating with a laser power of 3.3 kW fluctuates around 0.6, whereas it fluctuates around 0.42 for the substrate and the coating with a laser power of 3.7 kW. These fluctuations are usually attributed to the accumulation and removal of wear chips during friction. During wear, agglomeration makes the wear surface rougher, thereby increasing the coefficient of friction. Specifically, the accumulation of wear debris roughens the wear surface, which, in turn, triggers an increase in the coefficient of friction. This is because the accumulation of wear debris increases the roughness of the friction interface, leading to an increase in friction.

For the coating with a laser power of 3.3 kW, due to the low-energy-density laser in the laser melting process, the crystallinity of the grains in the coating decreases, and the strength of the solid solution phase produced is lower; at the same time, the friction coefficient of the coating increases due to the low-energy-density laser and the increased proportion of non-melting alloy powder in the coating, and the inhomogeneity of the coating organization. The average coefficients of friction of the TC4 substrate and the coatings with laser powers of 3.3 kW, 3.5 kW, and 3.7 kW were approximately 0.58, 0.40, 0.42, and 0.42, respectively. The coating with a laser power of 3.5 kW had the lowest average coefficient of friction and was stable, which indicates that it has the best wear resistance.

According to Archard′s wear law [34], the wear rate equation is as follows:

(6) $$K=\frac{V}{N\cdot d}$$

where *K*, *N*, *V*, and *d* represent wear rate, applied load (N), wear volume (mm^3^), and total sliding distance (m), respectively. The reciprocal of the wear rate is the wear resistance.

As shown in Figure 7c, the highest volumetric wear of 6.09 × 10^7^ μm^3^ was observed for the TC4 substrate, while the wear volumetric losses for coatings with laser powers of 3.3 kW, 3.5 kW, and 3.7 kW were 3.28 × 10^7^ μm^3^, 1.75 × 10^7^ μm^3^, and 2.85 × 10^7^ μm^3^, respectively. As the laser power increases, the amount of coating wear first decreases and then tends to increase. This is mainly due to the fact that the microhardness of the coatings increases and then decreases as the laser power increases, resulting in coatings with laser powers of 3.3 kW and 3.7 kW being susceptible to adhesive wear, which leads to material transfer between the friction surfaces, which, in turn, results in increased wear in localized areas, thus decreasing the wear resistance of the coatings. The calculated results of wear rate and abrasion resistance are shown in Figure 7d. The wear resistance of the coatings with laser powers of 3.3 kW, 3.5 kW, and 3.7 kW is 1.83, 3.42, and 2.13 times higher than that of the TC4 substrate, respectively, and the coating with laser power of 3.5 kW has the lowest wear rate. This experimental result shows that the hardness and wear resistance of the WNbMoTaV RHEA coating can be effectively improved by precisely regulating the laser power during the laser cladding process, which effectively improves the hardness and friction and wear properties of the TC4 titanium alloy surface.

**Figure 8:**
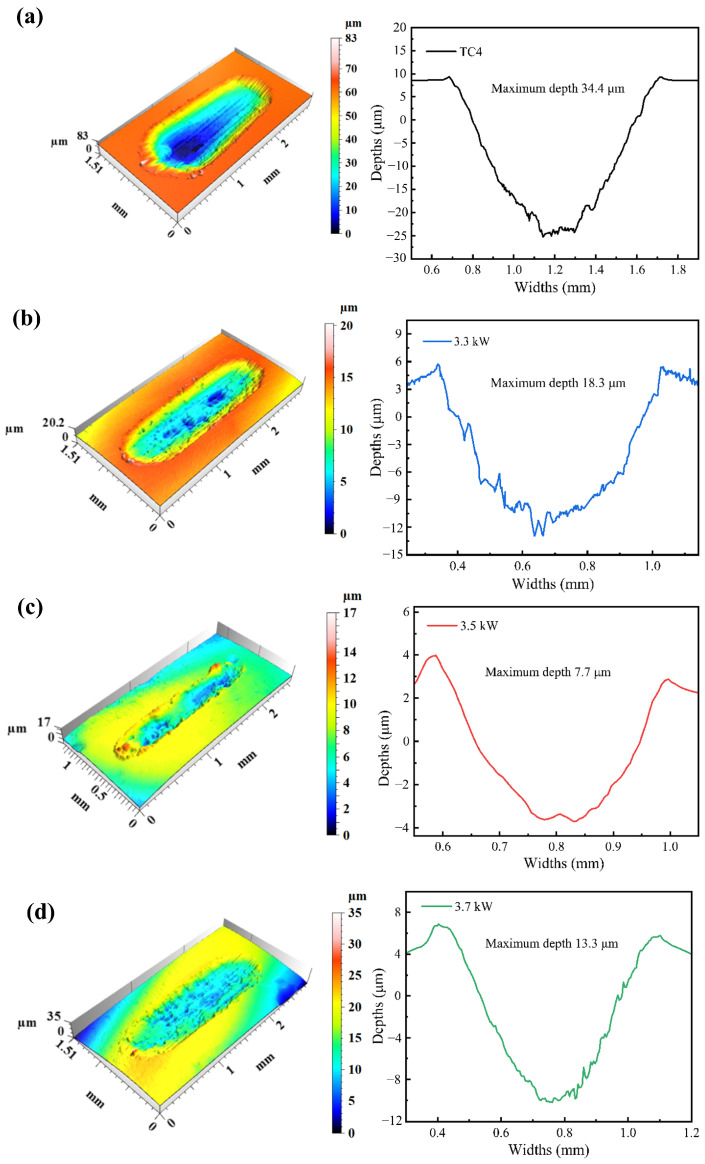
Three-dimensional morphology and cross-sectional profiles of TC4 substrate and coating wear marks: (**a**) TC4 substrate; (**b**) 3.3 kW; (**c**) 3.5 kW; (**d**) 3.7 kW.

The macroscopic morphology of the wear surfaces was analyzed in 3D. Figure 8 shows the 3D morphology and cross-section curves of the corresponding wear marks for the TC4 substrate and the coatings with laser powers of 3.3 kW, 3.5 kW, and 3.7 kW. The chromatic aberration in the image shows the depth variation of the worn surface; the amount of wear of the coating can be obtained from the 3D wear profile, the lines of the same color represent the furrows, and it can be clearly observed that the depth of the grooves of the TC4 substrate during friction wear is uniform and deep. The Si_3_N_4_ grinding balls pressed deeply into the TC4 surface, which exacerbated the wear volume loss due to plastic removal under reciprocating friction, resulting in the formation of wear marks with a maximum depth of about 34.4 μm. The maximum depths of wear marks for the WNbMoTaV RHEA coatings were 18.3 μm, 7.7 μm, and 13.3 μm for laser powers of 3.3 kW, 3.5 kW, and 3.7 kW WNbMoTaV RHEA coatings, respectively, as compared with the TC4 substrate. WNbMoTaV RHEA coatings showed shallower and narrower wear marks. The macroscopic narrowing of the wear mark area indicates a smaller true-contact area between the coating and the rubbing pair, which is related to the microhardness of the coating itself. Therefore, if the true-contact area becomes smaller and is accompanied by a lower wear rate, this indicates better wear resistance of the coating.

The cross-sectional width, depth, and wear area of the coatings with laser powers of 3.3 kW, 3.5 kW, and 3.7 kW are smaller than those of the substrate. This is mainly due to the high-strength BCC phase produced during the laser melting process and the rapid cooling to strengthen the grain boundaries, which can effectively resist the high-speed friction of Si_3_N_4_ grinding ball, indicating that the coatings have good wear resistance. For the coating with a laser power of 3.3 kW, it was the largest of the three coatings in terms of cross-section width and depth, which was mainly due to the low laser power, the high amount of unfused powder in the coating, and the lower laser power led to the generation of defects on the surface of the coating, which resulted in the lowest microhardness, and, therefore, deepened the depth of the abrasion marks. The increase in the depth of the abrasion marks is a direct response to the increased removal of material from the surface of the friction pair, i.e., the wear is increasing, indicating that the plastic deformation resistance of the coating has become slightly insufficient. The coating with a laser power of 3.5 kW has the best wear resistance, which is mainly attributed to the higher resistance to plastic deformation due to the production of more BCC phases with high strength during the laser melting process at this laser power, as well as fewer low-hardness matrix phases in the coating. The maximum width and depth of the coating were elevated with a laser power of 3.7 kW compared to the abrasion of the coating with a laser power of 3.5 kW. The deepening of the abrasion depth of the coating can be explained by the surface quality of the coating, which shows over-burning, indicating that the quality of the generated high-strength BCC phase is not as good as that of the coating with a laser power of 3.5 kW. The successive damage of the BCC phase in the over-burning reduces its supporting role, and, therefore, the abrasion marks deepen.

### 3.4. Wear Mechanisms

**Figure 9:**
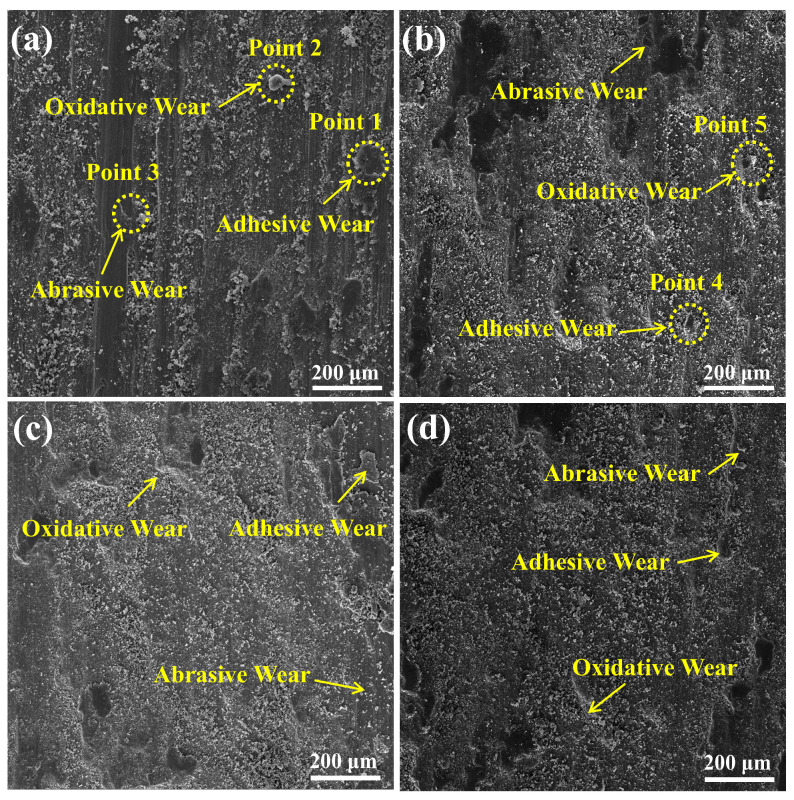
SEM images of the wear surface of TC4 substrate and WNbMoTaV RHEA coating: (**a**) TC4 substrate; (**b**) 3.3 KW; (**c**) 3.5 kW; (**d**) 3.7 kW.

Figure 9 shows the wear surface SEM images of the TC4 substrate and coating. Figure 9a shows the wear surface morphology of the TC4 substrate. The wear surface of the substrate exhibits significant plastic deformation and a large number of wide and deep grooves appear on the grinding surface. The results show that severe abrasive wear of the substrate occurs under the combined effect of oxides and abrasive debris, leading to significant friction in the grinding balls. Due to the low microhardness, the wear surface of the substrate becomes rough and the hard micro-convex body can easily penetrate the sliding surface of the substrate. Surface plowing occurs under a combination of high-pressure stress and shear stress. As the friction time increases, the surface material of the substrate is sheared and damaged, leading to separation of the surface material, formation of wear particles, and abrasive wear. In addition, there is adhesive wear on the wear surface of the substrate. The elemental distribution on the worn surface was analyzed using EDS and the results are shown in Table 3. The elemental composition consists mainly of O and Ti, indicating that oxidized wear has occurred on the substrate surface. Therefore, the wear mechanisms of the substrate are adhesive wear, oxidative wear, and abrasive wear.

The abraded surface morphology of the coating is shown in Figure 9b–d. It was observed that there was slight peeling in localized areas of the worn surface of the coating, however, a large amount of the coating remained bonded to the substrate and was not worn away. This is due to the formation of a high-strength BCC phase during the laser cladding process, which significantly reduces the plowing effect of the coating. The excellent wear resistance of the coating is mainly due to its high surface microhardness and resistance to plastic deformation. All three coating surfaces have only a small number of microscopic pear-shaped grooves, and the wear mechanism is slight abrasive wear. The wear pattern of the coating with laser power of 3.3 kW exhibited the largest plastic deformation as compared to the wear pattern of the coatings with laser powers of 3.5 kW and 3.7 kW, which was attributed to the higher amount of unfused powder and more coating defects in the coating with laser power of 3.3 kW, which resulted in the susceptibility of this coating to adhesive wear. The wear morphology of the coating with a laser power of 3.7 kW shows a larger plastic deformation than that of the coating with a laser power of 3.5 kW, which is mainly due to the successive damage of the medium- and high-strength BCC phase of the coating with a swept laser power of 3.7 kW, which reduces its supporting effect. The wear morphology of the coating with a laser power of 3.7 kW shows a larger plastic deformation than the wear morphology of the coating with a laser power of 3.5 kW, mainly due to the continuous damage of the high-strength BCC phase of the coating with a laser power of 3.7 kW, which reduces its supporting effect. Overall, the abrasive wear characteristics in the coating became less pronounced compared to the TC4 substrate. The EDS data in Table 3 show that the Si content at point 5 is about 4.3 wt.%. The Si element exhibits a localized bias on the coating surface, which may be due to the adhesion of abrasive particles to the coating surface during the friction process. Analysis of the elemental distribution on the wear surface of the coating using EDS reveals a large amount of O elements, indicating that the wear surface is oxidized by the frictional heat, leading to oxidative wear, which, in turn, provides protection by forming an oxide layer on the wear surface that effectively isolates the friction partner from the coating. Therefore, the main mechanisms of coating wear are oxidative wear, slight abrasive wear, and adhesive wear.

## 4. Conclusions

In this study, WNbMoTaV RHEA spherical alloy powder was prepared by radiofrequency plasma spheronization, and the sphericity, particle size distribution, and physical-phase composition of the prepared powder was investigated. Finally, the prepared WNbMoTaV RHEA spherical alloy powder was successfully prepared as a coating on the surface of TC4 titanium alloy by laser cladding technique. An in-depth study of the macrostructure, microstructure, microhardness, and wear resistance of the RHEA coating was performed, and the wear mechanism of the RHEA coating and TC4 substrate was analyzed. The following conclusions are summarized:

- The parameter settings of binder, radiofrequency power, and sheath gas are very important when preparing WNbMoTaV RHEA spherical alloy powder. Through the optimization of process parameters, the granulated powder showed high homogeneity; the binder system effectively ensured the integrity of the particles, and the alloy powder had excellent sphericity without any fragmentation or oxidation; the particle size distribution of the alloy powder was 15–45 μm, and the alloy showed a single BCC solid solution structure.
- For the prepared WNbMoTaV RHEA coatings, the macroscopic organization of the coatings showed no obvious defects. The percentage of unfused alloy powder coated with laser powers of 3.3 kW, 3.5 kW, and 3.7 kW was 19.3%, 11.2%, and 7.8%, respectively, and the dilution rates were 22.04%, 26.90%, and 32.27%, respectively. The coating is mainly composed of BCC phase and a Ti-rich phase. At a laser power of 3.7 kW, more Ti-rich phases are generated in the coating, and the crystallinity of the coating increases with the increase in laser power. The grain sizes of the coatings with laser powers of 3.3 kW, 3.5 kW, and 3.7 kW are 21.55 nm, 16.48 nm, and 16.17 nm, respectively. Higher laser power results in smaller grain sizes.
- The hardness of the coatings with 3.3 kW, 3.5 kW, and 3.7 kW was 1.72, 1.97, and 1.76 times higher than that of the substrate, respectively. In addition, the abrasion resistance of the coatings with laser powers of 3.3 kW, 3.5 kW, and 3.7 kW was 1.83, 3.42, and 2.13 times higher than that of the TC4 substrate, respectively. The main mechanisms of coating wear are oxidative wear, minor abrasive wear, and adhesive wear. The coating with the highest hardness and wear resistance is the 3.5 kW coating. Precise control of the laser power inhibits plastic deformation of the coating, thus increasing the wear resistance of the coating. This has a positive effect on the future application of TC4 titanium alloys in various wear parts in the field of precision military and aerospace.

## Figures and Tables

**Table 1:** Composition of alloy powder (wt.%).

Ta	W	Mo	Nb	V
31.7	29.2	16.3	16.2	6.6

**Table 2:** Chemical composition (wt.%) of the points selected from Figure 6.

Point	Mo	Nb	Ta	W	V	Ti
1	9.8	19.6	29.7	29.2	4.2	7.5
2	13.2	14.4	25.6	26.2	6.0	14.6
3	8.0	11.4	17.3	8.6	5.2	49.4

**Table 3:** Chemical composition (wt.%) of the points selected from Figure 9.

Point	Ti	O	Al	Si	N	W	Mo	Nb	Ta	V
1	28.5	50.5	2.5	12.6	4.8	-	-	-	-	1.1
2	60.8	31.2	5.5	-	-	-	-	-	-	2.5
3	87.0	6.2	4.8	-	-	-	-	-	-	2.0
4	3.7	28.4	-	5.2	12.2	15.3	7.9	7.2	17.4	2.7
5	2.1	52.6	-	4.3	1.6	12.2	5.9	5.7	14.8	0.8

## Data Availability

The original contributions presented in the study are included in the article, further inquiries can be directed to the corresponding author.

## References

[1] Li Y., Zhou Z., He Y. (2024). Tribocorrosion and Surface Protection Technology of Titanium Alloys: A Review. Materials.

[2] Luo H., Li J.b., Ye J.L., Tan J., Rashad M., Chen X.H., Han S.L., Zheng K.H., Zhao T.T., Pan F.S. (2022). Effect of Ti-6Al-4V particle reinforcements on mechanical properties of Mg-9Al-1Zn alloy. Trans. Nonferrous Met. Soc. China.

[3] Shi Z.M., Pang M. (2023). Study on the performance of laser nitriding in situ enhanced Co12/TC4 composite Ag self-lubricating coating on TC4 surface. J. Mater. Sci..

[4] Liu K., Yan H., Zhang P., Zhao J., Yu Z., Lu Q. (2020). Wear Behaviors of TiN/WS_2_ + hBN/NiCrBSi Self-Lubricating Composite Coatings on TC4 Alloy by Laser Cladding. Coatings.

[5] Gangwar K., Ramulu M. (2018). Friction stir welding of titanium alloys: A review. Mater. Des..

[6] Jin L., Li P., Zhou H., Zhang W., Zhou G., Wang C. (2015). Improving thermal insulation of TC4 using YSZ-based coating and SiO_2_ aerogel. Prog. Nat. Sci..

[7] Almonti D., Salvi D., Ucciardello N., Vesco S. (2024). Enhanced Wear Resistance and Thermal Dissipation of Copper-Graphene Composite Coatings via Pulsed Electrodeposition for Circuit Breaker Applications. Materials.

[8] Almonti D., Baiocco G., Millia M.D., Mingione E., Menna E., Rubino G., Salvi D., Stamopoulos A., Ucciardello N. (2024). Morphological and functional characterization of electroplated Ni-graphene composite coatings. J. Phys. Conf. Ser..

[9] Peta K., Bartkowiak T., Rybicki M., Galek P., Mendak M., Wieczorowski M., Brown C.A. (2024). Scale-dependent wetting behavior of bioinspired lubricants on electrical discharge machined Ti6Al4V surfaces. Tribol. Int..

[10] Atroshenko S.A., Valiev R.Z., Morozov N.F., Valiev R.R., Savina Y.N., Antonova M.N., Evstifeev A.D. (2024). Wear and failure analysis of Ti-6Al-4V titanium alloy with a protective coating during high-speed erosion. Phys. Mesomech..

[11] Lv Y.H., Li J., Tao Y.F., Hu L.F. (2016). Oxidation behaviors of the TiNi/Ti_2_Ni matrix composite coatings with different contents of TaC addition fabricated on Ti6Al4V by laser cladding. J. Alloys Compd..

[12] Li W., Liu P., Liaw P.K. (2018). Microstructures and properties of high-entropy alloy films and coatings: A review. Mater. Res. Lett..

[13] Weng F., Chen C., Yu H. (2014). Research status of laser cladding on titanium and its alloys: A review. Mater. Des..

[14] Candel J.J., Amigó V., Ramos J.A. (2010). Sliding wear resistance of TiCp reinforced titaniumcomposite coating produced by laser cladding. Surf. Coat. Technol..

[15] Yeh J.W., Chen S.K., Lin S.J., Gan J.Y., Chin T.S., Shun T.T., Tsau C.H., Chang S.Y. (2004). Nanostructured high-entropy alloys with multiple principal elements: Novel alloy design concepts and outcomes. Adv. Eng. Mater..

[16] Cui K., Zhang Y. (2023). High-entropy alloy films. Coatings.

[17] Wang Y., Yang Y., Yang H. (2018). Microstructure and wear properties of nitrided AlCoCrFeNi high-entropy alloy. Mater. Chem. Phys..

[18] Li J.H., Tsai M.H. (2020). Theories for predicting simple solid solution high-entropy alloys: Classification, accuracy, and important factors impacting accuracy. Scr. Mater..

[19] Xie X., Li N., Liu W. (2022). Research progress of refractory high entropy alloys: A review. Chin. J. Mech. Eng..

[20] Zhang B., Huang Y., Dou Z., Wang J., Huang Z. (2024). Refractory high-entropy alloys fabricated by powder metallurgy: Progress, challenges and opportunities. J. Sci..

[21] Miracle D.B., Senkov O.N. (2017). A critical review of high entropy alloys and related concepts. Acta Mater..

[22] Jiang L., Hu Y.J., Sun K. (2020). Irradiation-induced extremes create hierarchical face-/body-centered-cubic phases in nanostructured high entropy alloys. Adv. Mater..

[23] Ren Z.Y., Hu Y.L., Tong Y. (2023). Wear-resistant NbMoTaWTi high entropy alloy coating prepared by laser cladding on TC4 titanium alloy. Tribol. Int..

[24] Hao X., Liu H., Zhang X., Tao J., Wang Y., Yang C., Liu Y. (2023). Microstructure and wear resistance of in-situ TiN/(Nb, Ti)_5_Si_3_ reinforced MoNbTaWTi-based refractory high entropy alloy composite coatings by laser cladding. Appl. Surf. Sci..

[25] Senkov O.N., Wilks G.B., Scott J.M., Miracle D.B. (2011). Mechanical properties of Nb_25_Mo_25_Ta_25_W_25_ and V_20_Nb_20_Mo_20_Ta_20_W_20_ refractory high entropy alloys. Intermetallics.

[26] Liu B., Duan H., Li L. (2021). Microstructure and mechanical properties of ultra-hard spherical refractory high-entropy alloy powders fabricated by plasma spheroidization. Powder Technol..

[27] Na T.W., Park K.B., Lee S.Y. (2020). Preparation of spherical TaNbHfZrTi high-entropy alloy powders by a hydrogenation-dehydrogenation reaction and thermal plasma treatment. J. Alloys Compd..

[28] Xia M., Chen Y., Chen K. (2022). Synthesis of WTaMoNbZr refractory high-entropy alloy powder by plasma spheroidization process for additive manufacturing. J. Alloys Compd..

[29] Jiang Y.Q., Li J., Juan Y.F. (2019). Evolution in microstructure and corrosion behavior of AlCoCr_x_FeNi high-entropy alloy coatings fabricated by laser cladding. J. Alloys Compd..

[30] Lipson H. (1979). Elements of X-ray diffraction. Contemp. Phys..

[31] Shao J.Z., Li J., Song R., Bai L.L., Chen J.L., Qu C.C. (2020). Microstructure and wear behaviors of TiB/TiC reinforced Ti_2_Ni/α(Ti) matrix coating produced by laser cladding. Rare Metals.

[32] Zhao Y., Ma M., Huang C., Lin M., Tu J., Wang H., Zhan Z., Liu H., Chang X., Duan H. (2024). Microstructure, high temperature wear resistance and corrosion behaviour of NiCrCoNbMo_x_ high-entropy alloy coatings on 15CrMoG alloy by laser cladding. Mater. Today Commun..

[33] Wang C., Gao Y., Wang R., Wei D., Cai M., Fu Y. (2018). Microstructure of laser-clad Ni60 cladding layers added with different amounts of rare-earth oxides on 6063 Al alloys. J. Alloys Compd..

[34] Archard J.F. (1953). Contact and rubbing of flat surfaces. J. Appl. Phys..

